# The Almoner Question

**Published:** 1921-03-19

**Authors:** 


The Hospital

The Workers' Newspaper of Administrative Medicine and Institutional Life. Administration. National Insurance and Health.

No. 1815. Vol. LXIX. SATURDAY, MARCH 19, 1921. PRICE TWOPENCE

### THE ALMONER QUESTION.

While we cannot go so far as a distinguished journalist, who privately asserts that the *Daily Mail* is a newspaper written by women for women, we agree that it does sometimes jump to conclusions in a startling way. The latest example is its panegyric of the lady almoner. At a time when praise and encouragement were needed—namely, when the Almoners' departments were first formed—we were not slack to point out that the prevention of hospital abuse was only one of the almoner's duties, and that after-care work, far beyond the limited scope of the old Samaritan Funds, should increasingly occupy her attention. The two sides, and the several duties, were admirably outlined in some articles by Miss Cummins, the distinguished almoner at St. Thomas's Hospital, which appeared in our columns in July 1917. To-day events incline the department to veer in the opposite direction. There is, and rightly, a Hospital Almoners' Council, the duty of which, we take it, is to direct the training of almoners, to maintain a higher standard of work, and to encourage loyalty not only to itself, but to the hospitals for whom the almoners were called into being. Encouragement of the work by protection of the work through the workers is the proper province of such a body. But protection can be overdone, and it will be overdone, should protection be invoked, not on behalf of the standard of the work and its remuneration, but against the authorities under whom the work has to be done. It is natural that such bodies should keep files of vacancies and the like, and seek to encourage in every legitimate way the interests of their qualified members. But it would be a mistake for the members to rely too exclusively on the hopes held out by these bodies. For example, no hospital need be bound to apply, say, to the Hospital Almoners' Council when seeking to fill a vacancy. It is rather the duty of the Council to apprise the hospitals of suitably trained candidates, and then, having helped both sides as far as possible, to let competition take its customary course. For of this we may be sure, that while professional organisations and the hospitals are necessary to each other, and while co-operation can be of the greatest help to both, if the latter are antagonised by undue assertiveness, they can retaliate by rejecting candidates who are presented in improper form.

The matter needs attention at the present moment, because the work of the almoner has now passed into a new set of conditions. They originated to prevent hospital abuse, and were friendly inquiry agents. Their inquiries led them, and rightly, not only into the antecedents of the patients, but into their prospects and impending history after leaving the institution. Then came the war, and with it the new attempt to organise contributions from all classes, including the patients themselves. The administrative effect of this work, which was naturally largely thrown upon the almoners, was greatly to add to the hospital secretaries' and the almoners' correspondence. This correspondence requires very delicate handling, handling so delicate that no single person should take upon himself the responsibility of dealing with it without consultation, for more than one department will, directly or indirectly, suffer if an indiscretion shall occur. Now the head of all the administrative departments is the hospital secretary, and his counsel in cases of difficulty should be invaluable to his departmental chiefs. All the administrative threads meet in the secretary's office. He is the nodal point, and therefore must be in a better position than any other single officer to view the whole position. For the duty of a superintendent is to superintend, and to secure harmonious work by the balance of each administrative department with every other. Thus, if an almoner should be indiscreet and decide a delicate matter without consideration, her offended correspondent will decline to address himself to her, and apply direct to the secretary. Such applications, of course, a secretary above all things wishes to avoid, because in the normal course too many things have to be referred to him as it is. His object is to make every department as independent as he can, but such independence has its complement in loyalty. No doubt, just as some secretaries are likely to be too strict, and some too lax, in their rulings, the unstable equilibrium which is the health of all living corporations is apt to lean too much on one side or the other. The Hospital has had to record biases in either direction. So long ago as 1913 the Metropolitan Hospital

Saturday Fund and the almoners were, for the moment, not at one, and in the early days some hospitals took, perhaps, a too conservative view of an almoner's duties. To-day, however, the almoner is "all the rage"; the bias is toward too great independence. This we regret, for the sake of the almoners themselves, since if they should display too much assertiveness they will cease to be valuable, and valueless officers do not survive.

Payments by patients provides the test-case. These payments began, perhaps, as a panic measure, and we may have to pay for that now by some dislocation of discipline. While there must be give-and-take on both sides, it yet remains true that, though the collection of such payments may be properly a departmental matter, the policy to be pursued concerning them is essentially an administrative one. As such, the secretary must have the deciding voice, because he is the Executive officer of the hospital Committee of Management. All other departmental chiefs must be affiliated under him, because he is the administrative superintendent, not of one department, but of all. Were this principle generously understood by all parties, the almoner's department would be free from certain anxieties which, in the main, they seem to have brought upon themselves, and from which, in the long run, they will be the principal sufferers. We therefore utter a word of warning in the belief that temperate counsels may yet prevail, and any misunderstanding frankly discussed with the object of dispersing them.

